# Retraction Note: Serum sphingosine-1-phosphate levels and *Sphingosine-1-Phosphate* gene polymorphisms in acute respiratory distress syndrome: a multicenter prospective study

**DOI:** 10.1186/s12967-022-03829-2

**Published:** 2022-12-21

**Authors:** Jiangnan Zhao, Yan Tan, Li Wang, Xin Su, Yi Shi

**Affiliations:** 1 Department of Respiratory and Critical Medicine, Jinling Hospital, Medical School of Nanjing University, No. 305 Zhongshan East Road, Nanjing, 210000 China; 2 Department of Respiratory Medicine, Nanjing First Hospital, Nanjing Medical University, Nanjing, 210000 China

**Retraction:J Transl Med (2020) 18:156** 10.1186/s12967-020-02322-y

The Editor-in-Chief has retracted this article. After publication concerns were raised about the nomenclature and methodology. Post-publication peer review has confirmed that the S1P (site 1 protease) gene does not encode the sphingolipid metabolite sphingosine-1-phosphate. There is no gene for sphingosine-1-phosphate. As the study is based on sphingosine-1-phosphate gene polymorphism the Editor-in-Chief no longer has confidence in the results and conclusions presented. All authors agree with this retraction.

